# The ethnobotany and biogeography of wild vegetables in the Adriatic islands

**DOI:** 10.1186/s13002-019-0297-0

**Published:** 2019-03-29

**Authors:** Łukasz Łuczaj, Marija Jug-Dujaković, Katija Dolina, Mirjana Jeričević, Ivana Vitasović-Kosić

**Affiliations:** 1 Department of Botany, Faculty of Biotechnology, University of Rzeszów, ul. Pigonia 1, 35-310 Rzeszów, Poland; 2 Institute for Adriatic Crops and Karst Reclamation, Put Duilova 11, 21000 Split, Croatia; 3 Institute for Marine and Coastal Research, University of Dubrovnik, Kneza Damjana Jude 12, PO Box 83, 20000 Dubrovnik, Croatia; 4 Žrnovo, Croatia; 5 Department of Agricultural Botany, Faculty of Agriculture, University of Zagreb, Svetošimunska cesta 25, 10000 Zagreb, Croatia

**Keywords:** Wild edible plants, Wild food plants, Ethnobiology, Leafy vegetables, Mediterranean diet

## Abstract

**Background:**

Archipelagos of islands have played an important role in shaping some of the paradigms of biology, including the theory of the evolution of species. Later, their importance in biology was further emphasised by the theory of island biogeography, which contributed to a better understanding of the shaping of species richness not only on real islands, but on isolated habitat islands as well. Although ethnobotany is a well-established discipline, patterns of knowledge about plant uses in archipelagos have never been quantitatively analysed, and the whole concept has been only briefly mentioned in the ethnobiological context.

The aim of our study was to record which taxa of wild vegetables have been consumed in the Adriatic islands and to establish if such variables as island size, population size, flora or its isolation are correlated with the number of wild vegetables used.

**Methods:**

We interviewed 225 people (15 from each island).

**Results:**

Altogether, the use of 89 species of wild vegetables has been recorded. The largest number of wild vegetables is eaten on the islands of Korčula, Vis and Šolta, and the lowest on Ugljan, Cres and Dugi Otok. The studied independent variables had a small and statistically not significant effect on the wild vegetable list length. The most visible effect was an increasing trend from north-west to south-east, overrunning the typical biogeographical island patterns. Moreover, one of the large and well-populated islands, Korčula, showed an ‘unusually’ high level of wild vegetable use. We hypothesise that the current use of so many species on this island has been maintained by the inhabitants’ awareness that they are the holders of relic knowledge, an awareness reiterated by ethnographic and popular publications, as well as a strong history of famine. The most interesting edible species used in the Adriatic islands are *Bunium alpinum*, *Cytinus hypocystis* (both mainly on Pašman), *Lotus edulis* (on Vis) and *Posidonia oceanica* (on Vis and Korčula).

**Conclusions:**

The recorded relationships between the demographic and geographical features of the islands were statistically not significant. We assume that cultural and historical factors diversifying the use of plants in particular islands are stronger than the above-mentioned measurable variables.

## Background

Archipelagos of islands have played an important role in shaping some of the paradigms of biology, including the theory of the evolution of species created by Charles Darwin and Alfred Russel Wallace [1]. The former biologist developed it by the study of the features of closely related species in the Galapagos Islands and the latter by the research in the islands of present-day Indonesia. Later, their importance in biology was further emphasised by the theory of island biogeography created by Robert MacArthur and Edward O. Wilson [2]. This theory helps us understand the shaping of species richness not only on real islands, but on isolated habitat islands as well. Although ethnobotany is a well-established discipline, patterns of knowledge about plant uses in archipelagos have never been quantitatively analysed. The whole concept has been only briefly mentioned in the ethnobiological context [3], in spite of the fact that several ethnobotanical studies have qualitatively compared the uses of plants on groups of islands, particularly in Polynesia (e.g. [4, 5]). In our paper, we look at the species richness of wild vegetables used by the inhabitants of the 15 largest Adriatic islands in Croatia.

The reason island biogeography theory has not been tested in ethnobotany stems from the extreme complexity of the relationship between humans and plants. For instance, two communities using similar resources and living in the same or neighbouring areas may differ in plant use [6]. Moreover, humans migrate, and it would take very isolated islands and low technology to keep most human individuals from ever leaving their ancestral island. We should, however, keep in mind that living on islands might sometimes limit migration and reduce the exchange of human knowledge. The communities on the Eastern Adriatic Islands in Dalmatia, Croatia, which are the object of our study, have been extensively studied anthropologically (e.g. [7–10]). The people living on islands more remote from the coast display an unusually high degree of genetic isolation, endogamy and inbreeding, and even now, in the times of tourism, the percentage of indigenous island population varies between 70 and 98% [10]. Thus, most inhabitants, even if they have travelled or lived outside the Adriatic islands, are usually associated with one island within the researched area. The aim of our study was to find out whether island characteristics had some influence on plant use patterns.

The area of our study covered the Croatian islands of the Adriatic coast. Of the 718 islands, only 47 are inhabited, in the sense that at least one person resides on that island [11]. However, many of those ‘permanent’ inhabitants are people who have emigrated to the mainland and draw tax benefits from being registered as island inhabitants: they visit the island only during summer, or even only every few years. Thus, the number of year-round inhabitants is well under half of the official total. The net population growth in most of the islands is negative, and the population of the islands has declined by 30% since a century ago [12].

Most of the larger Croatian islands have had their vascular floras described in detail [13, 14] and have been the subjects of biogeographical analyses [13].

We chose wild vegetables as the studied domain of knowledge. Their use used to be widespread in Mediterranean agroecosystems but is now declining due to changes in modern diets and lifestyles and the intensification of agriculture [15–18]. This also holds true for a few of the coastal areas on mainland Croatia and Herzegovina that we studied previously, as well as the island of Krk [19–25]. Using wild vegetables can be seen as one of the typical features of the Mediterranean cuisine and lifestyle [18, 26], and it has been highlighted that the use of numerous species of wild vegetables is more common in the south than in the temperate parts of central and northern Europe.

Up until recently, the gathering of wild vegetables had mainly been a domain of traditional knowledge passed down within families, little-influenced by literature, in contrast to ethnomedicinal knowledge, which is highly influenced by old and new texts and other media [27]. For local inhabitants, wild vegetables are a well-defined cognitive domain, and, according to our observations, the distinction between wild and domesticated greens is clearer than in the case of fruits.

The aim of the study was:

1. To record which taxa of wild vegetables have been consumed in the Adriatic islands
2. To establish if such variables as island size, population size, flora or its isolation are correlated with the number of wild vegetables used

We made a hypothesis that the length of the total wild vegetable list per island, as well as the median number of species per informant, is positively correlated with:

1. The number of species reported in the floras of specific islands. The link between the flora and plant use is obvious: the more species available, the more likely it is that more species are used.
2. The area of the island. A larger area within which interviews were carried out meant a larger chance for different species to be found as well as a smaller similarity in village traditions due to the larger physical distance between villages.
3. The number of inhabitants. The more people live on the island, the more exchange of knowledge is likely to happen and there are more knowledge holders.
4. The proximity of mainland (i.e. is negatively correlated with the distance from the mainland of Croatia). We assumed that in less isolated islands, whose inhabitants have more social contacts with the mainland, there is more opportunity for the exchange of knowledge.

The hypotheses no. 2 and 4 are directly testing the island biogeography theory [2] and no. 1 and 3 result from it indirectly.

## Methods

**Fig. 1:**
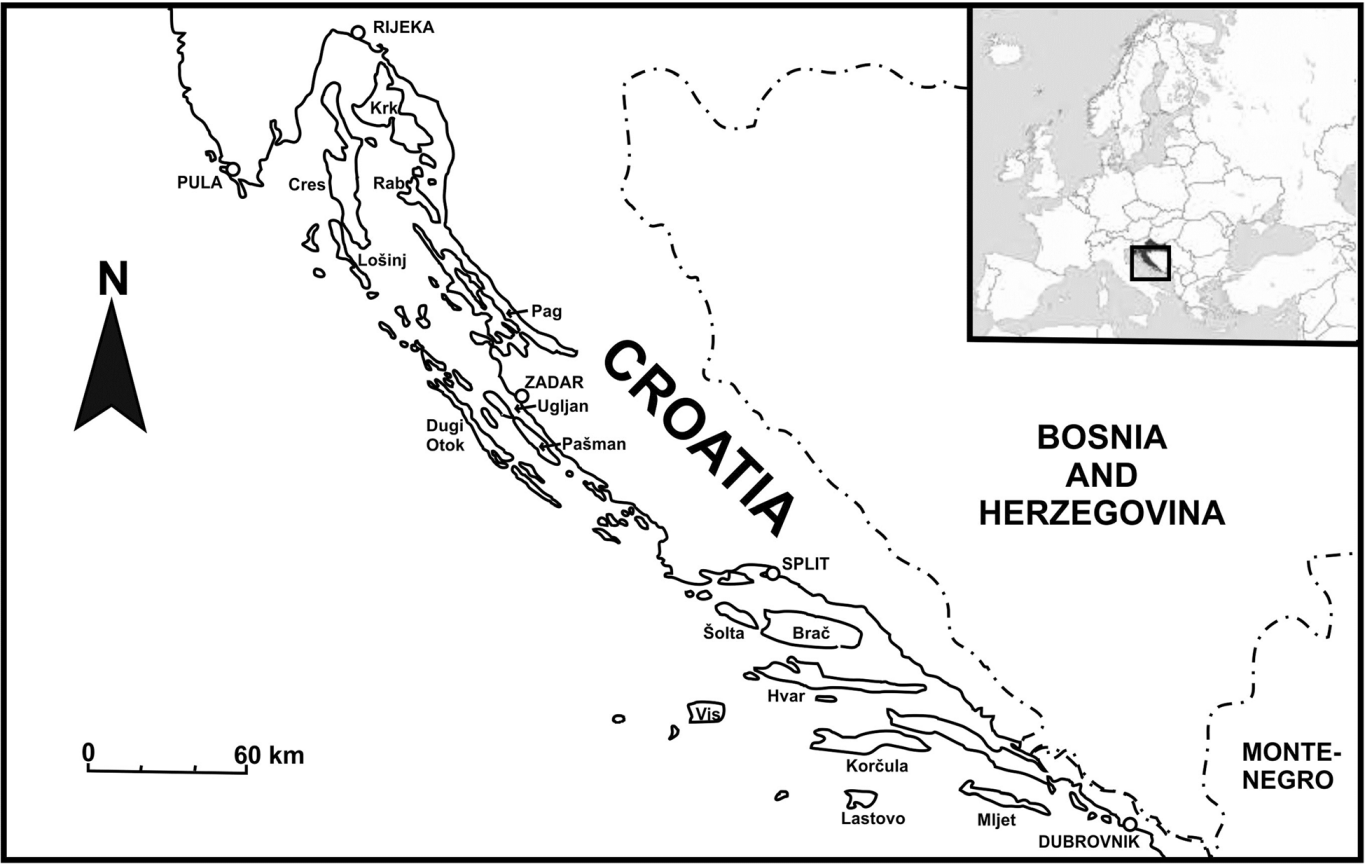
Map of the Adriatic Sea and southern Croatia showing the studied islands (normal font) and major cities (in capitals)

We selected the 15 largest islands, those with an area > 40 km^2^ (Table 1, Fig. 1). The study was performed between 2013 and 2018, with most interviews conducted in 2016 and 2017, in seasons when wild vegetables can be found (spring or autumn). We applied the classic methods of ethnobotany [28–31]: in-depth semi-structured interviews starting from freelisting and supplemented, if possible, by walks around the places where the respondents gathered plants and could identify the supplied names. On each island, we interviewed 15 key informants (those who know and collect wild foods) recommended by inhabitants, villages leaders, etc. Some key informants were also selected from people found working in the fields and claiming that they still collected wild food plants. The interviews were performed in Croatian, the native language of the inhabitants. The interviews concerned different aspects of plant use, but here, we present data only about wild vegetables. The general question of which ‘wild vegetables’ people used for food was supplemented with questions about ‘edible asparagus-like plants’ and wild vegetables preserved in vinegar, as some respondents tended to forget these plants when asked only about ‘wild vegetables.’ The category of asparagus-like plants is an emic one. It consists of plants whose young long shoots are eaten.

**Table 1:** Basic island statistics

	No. of wild vegetables	Median no. of vegetables	Area (km^2^)	Population	Flora	Longitude (° E)	Isolation (minimum km distance from mainland)
Brač	30	9	395	13,956	750	16.66	5
Cres	18	4	406	3079	1250	14.39	5
Dugi Otok	19	4	113	1655	540	15.03	16
Hvar	22	9	297	11,077	1046	16.8	4
Korčula	46	16	271	15,522	858	16.93	1
Krk	29	8	405	19,383	1170	14.61	0.8
Lastovo	21	8	41	792	678	16.87	26
Lošinj	26	8	74	7587	1300	14.43	29
Mljet	30	7	98	1088	712	17.55	8
Pag	31	9	284	9059	650	15.04	0.4
Pašman	26	7	60	2845	629	15.34	2
Rab	24	7	86	9328	800	14.77	2
Šolta	33	11	58	1700	267	16.31	15
Ugljan	18	8	51	6049	–	15.17	4
Vis	39	12	90	3445	598	16.16	43

We made efforts to cover the whole island evenly and recruit each informant from a different village (if the number of villages on an island was over 14). The number 15 was chosen as in less populated islands, it was difficult to recruit a larger number of key informants who actively gathered wild vegetables. We interviewed more women (65%) than men as they were usually identified by local informants as key informants; however, on each island, some male informants were also interviewed. The mean age of informants was 70. Key informants were chosen from people who were born on the islands and had their ancestry there.

The data for most islands has never been used before in any paper, but the data for Krk forms a subset of a larger set of interviews from this island performed for the comparison of historical and present uses of wild plants [21]. From this subset, we chose the first 15 interviews, which represented 15 villages.

The number of species in the islands’ floras was extracted from data gathered by Nikolić et al. [13], supplemented by the flora of Pašman [14]. The island’s isolation was measured as the distance (km) between the mainland and the part of the island closest to it. The population data were taken from the Statistical Yearbook as of 2015 [11].

Plants were identified using standard floras available in this area of Europe, including Domac’s guide for the identification of Croatian flora [32], Pignatti’s flora of Italy [33] and the Flora Croatica Database [34]. Plant names were updated to be consistent with the Plant List [35]. Voucher specimens were collected on the islands where they are used, usually with the assistance of the respondents. For deposition place, see the ‘Availability of data and materials*’* section of the paper.

Statistical analysis was performed using open access PAST software [36]. The significance and strength of the relationships between variables was assessed using correlation coefficients. The normality of distribution of variables was tested with the Shapiro-Wilk test. Most variables had normal distribution. Only the variable Isolation had to be log-transformed to achieve normality and the variable Area did not become normal even after log-transformation. That is why for the latter variable, we applied the non-parametric Spearman rank correlation coefficient, whereas other variables were correlated using the parametric Pearson correlation coefficient. To visualise the similarity in wild vegetable species lists between the islands, and see whether this was associated with geographical proximity, we performed a detrended component analysis (DCA) on the species level [37]. We plotted the results of DCA on the two main axes that caused the distribution of the data to visualise potential overlap and variation in the species composition used in different islands. Another way of visualising the diversity of species composition on different islands was a numeral taxonomy dendrogram obtained by clustering. We applied the most commonly used method of clustering, i.e. unweighted pair group method with arithmetic mean (UPGMA), using Euclidean distance [38, 39].

## Results and discussion

**Fig. 2:**
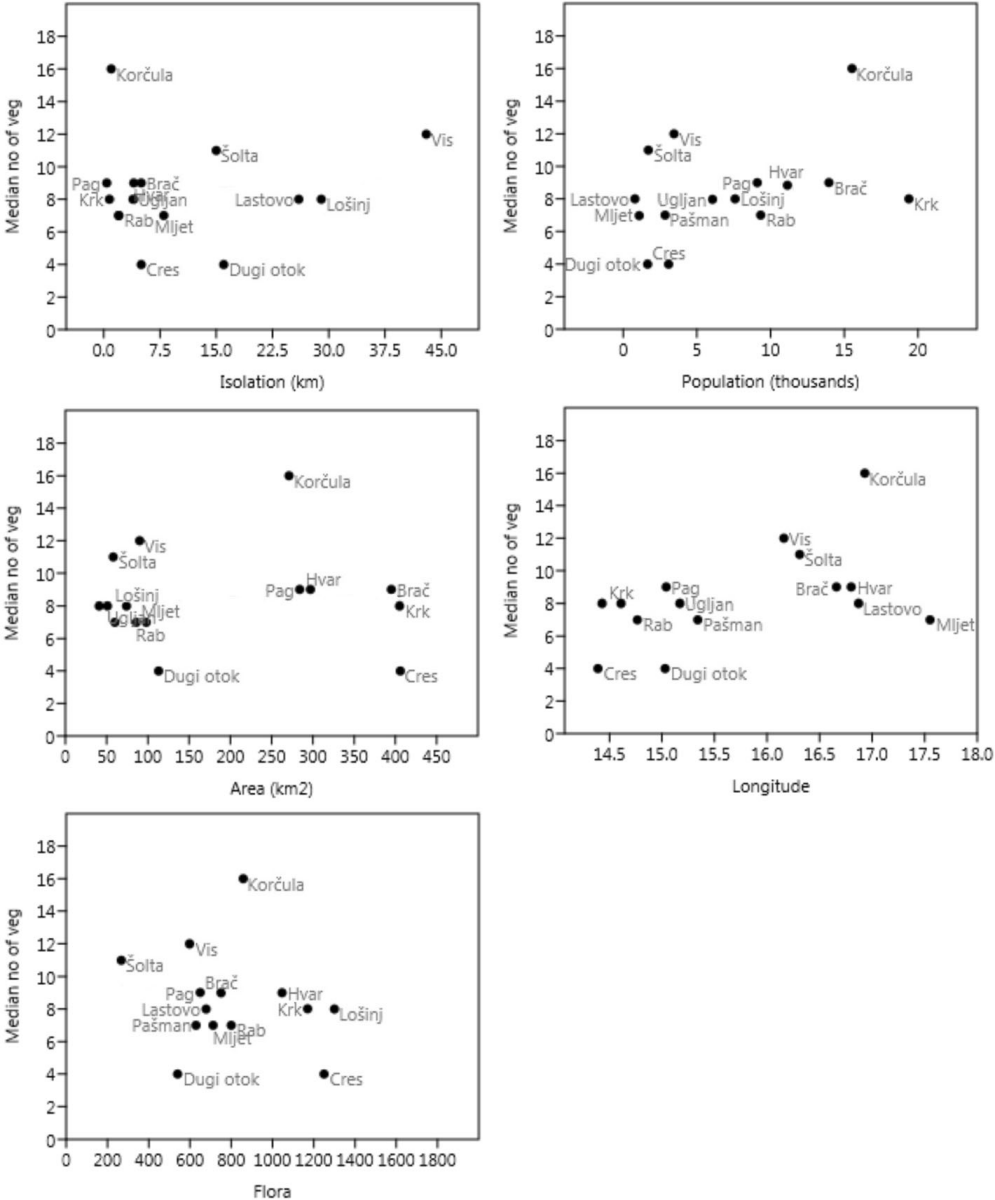
Scatterplots of the median numbers of vegetables used and the studied independent variables

Altogether, 89 taxa of wild vegetables from 31 plant families were identified to the species or genus level (Tables 2 and 3, Fig. 2). The longest lists of taxa used were found on Korčula (46 folk taxa), Vis (39) and Šolta (33). The shortest lists were found on Ugljan (18), Cres (18) and Dugi Otok (19). Korčula also had the highest median and mean number of species listed per interview (16). The best-represented families were Asteraceae (24 species), as well as Brassicaceae (9) and Apiaceae (8).

**Table 2:** Local names of wild vegetables

	Voucher no.	Part used	Preparation	Most common names
Alliaceae				
*Allium ampeloprasum* L.	WA0000066378	WH^a^	r/c	divlji luk, poriluk; also: pazduh LO, ljutica PG, lučac LO, lučić PG, RA, porić BR, paric VI, purić SO, BR, puriluk SO, HV, praska ML
other *Allium* spp. (mainly *Allium roseum* L.)	WA0000066454	WH	r/c	divji luk LO, KR, BR, divlji lučić KR, SO, jutika BR, ljutica PS, lučica KR, divlji češnjak KR
Amaranthaceae				
*Amaranthus* cf. *retroflexus* L.	ZAGR39998	L	c	šćirenica PG, SO, štir KO, PG, RA
*Beta vulgaris* L.	WA0000066322	L	c	divlja blitva (throughout); also: dibio blitva BR, divja blitva VI, šćav KR
*Chenopodium album* L.	WA0000066308	L	c	loboda; also: lobod KR, RA
*Salsola soda* L.	WA0000066392	L	c	rosica PG
*Sarcocornia fruticosa* (L.) A.J.Scott	WA0000066902	L	c	omaga LO, smucanj RA, smucalj RA
Apiaceae				
*Anethum graveolens* L.	WA0000066391	L	r/c	anit SO, anita PG, aniž SO, kopar LAS
*Apium graveolens* L.	WA0000066346	L	c	šelen PG
*Bunium alpinum* Waldst. & Kit. s.l.	WA0000066917	Root tubers	r	koprci BR, PS, koprcini PS
*Crithmum maritimum* L.	WA0000066324	L	m, also c	motar/matar; also: petrovac PG, DO, šćulac LO, ščulac PG, šćirenica KR, trova od mora VI
*Daucus carota* L.	WA0000066462	L	c	divlja mrkva; also mrkurela LA, mrkviej BR
*Foeniculum vulgare* Mill.	WA0000066401	L	r/c	komorač LO, DO, KO, ML, VI, koromac LO, VI, koromač BR, CR, DO, HV, KO, KR, LO, PG, PS, RA, SO, UG, VI, kromač KR, morač KO, ML, LA
*Smyrnium olusatrum* L.	WA0000066377	L	c	divlji selen LA, postolažina LA, postoložena LA
*Tordylium apulum* L.	WA0000066382	L	c	lembrc KO, vrati muž KO
Araceae				
*Arum italicum* Mill.	WA0000066915	L^a^	lb	arum CR, gujino zelje SO, kozlac LO, štarkavac CR, strtok KR, zminac DO, žuminac VI
Asparagaceae				
*Asparagus acutifolius* L.	WA0000066368	SH	c/r	šparoga, sparoga (throughout); also: asparadži CR, šparuga LO, šporovi CR
*Asparagus officinalis* L.	WA0000066906	SH	c/r	pitoma šparoga CR
*Leopoldia comosa* (L.) Parl.	WA0000066916	WH	c	fratar KR
*Ornithogalum* sp.		WH	c	–
*Ruscus aculeatus* L.	WA0000066369	SH	c	fraterska šparožina KR, kataroška KR, piturožka RA, pundži topo CR, rakže ML, koštrika ML, sjeskavica LA, veprina KR, LO, ML, veprinac LO
Asphodelaceae				
*Asphodelus aestivus* Brot.	WA0000066433	Root tubers^a^	lb	brden LO, cefarnjok VI
Asteraceae				
*Bellis* sp.		WH	c	tratinčica KO, VI
*Carduus pycnocephalus* L.	WA0000071128	L	c	drača SO, ošebad KO, osjak KO, oslobod VI, sikavac RA, sikavec PG, sikavica DO
*Chondrilla juncea* L.	WA0000071121	L	c	tavka PG
*Cichorium intybus* L.	WA0000066320	L	r/c	žutenica/žutinica/žutjenica (Dalmatian Islands); divlji radič/divlji radić (Kvarner Islands, LA, ML)
*Crepis dioscoridis* L.	WA00000	L	r/c	šćupej KO, žutinica KO, gorčica BR, gorcik VI, gorčik HV
*Crepis rubra* L.	WA0000066436	L	c	šćupej KO, šćjuper BR, šćuperuša BR
*Crepis sancta* (L.) Babc.	ZAGR9316	L	r/c	maslačak KR, RA, divlji radić RA, žutenica čupava KR
*Helminthotheca echioides* (L.) Holub.	WA0000066360	L	c	hrastavica KO, krastavica PS, lipavac PS, lipavica PS, prosenjica RA, rastej ML, tustoč BR, tustočel HV
*Lactuca perennis* L.		L	c	divlji špinat, modra salata BR
*Lactuca sativa* L. [feral]		L	r/c	pičola SO, HV, loćika KO
*Lactuca serriola* L.	WA0000066412	L	c	divlja salata; also: gorka salata BR, pasja salata VI
*Lactuca viminea* (L.) J. Presl & C.Presl	WA0000071123	L	c	gnjaška KO, nastriženica VI
*Leontodon tuberosus* L.	WA0000066329	L	c	korenjaška KO, also: grglava BR, lavji zub ML, podparuša ML, ugrin glava KO, undrglava KO
*Reichardia picroides* (L.) Roth	WA0000066328	L	r/c	antačola RA, natančola LO, RA, ratančola RA, barbaruša KR, berbečica PG, b(e) rberuša DO, PG, beršaka PG, bršača LO, bršljaka PG, ML, brusača KR, dušica BR, SO, iglica UG, jagla LO, jaglac LO, jogula LO, marta duha BR, matederica VI, materduh HR, materduha BR, HV, materinduh HV, matuderica VI, mojčinduh HV, slaška/slačka KO, sladić ML, tavka LA
*Rhagadiolus stellatus* (L.) Gaertn.	WA0000066445	L	c	kokošinja guzica, kokošinja guzica KO
*Scolymus hispanicus* L.	WA0000066345	L	c	brbeč PS, bremečica PG, brisača KR, brmeč KR, oščibod VI, sikavac PG, sisavica KO, skolub KO
*Scorzonera laciniata* Jacq.	WA0000071122	L	c	kozja brada HV, BR, KO, SO, kozja broda BR, kozjo brada VI, kozjo broda VI, kuzjo brada VI, kušnja broda VI
*Silybum marianum* (L.) Gaertn.	WA0000066349	L	c	beli trn KR
*Sonchus* spp.				blešnjak LO, bliješnjak LO, CR, blišnjak LO, blješnjak LO, blišnjak LO, PS, čepčeg/čevčeg ML, kostreč LA, kostric VI, kostrić/kostrič BR, HV, KO, LA, VI, kostriš DO, LO, UG, mišnjak PS, UG, mlič/mlić PG, mličac DO, mličak UG, mličnjak DO, PG, UG, mlišnjak UG, ostak/ostek CR, KR, sinjorac PG, RA, špilišor KR, šušak/sušak SO
*Sonchus asper* (L.) Hill	WA0000066913	L	c	
*Sonchus asper* subsp. *glaucescens* (Jord.) Ball ex Ball	WA0000066912	L	c	
*Sonchus oleraceus* L.	WA0000066305	L	c	
*Taraxacum* sp.	WA0000066372	L	r/c	maslačak (throughout), also: paric VI, retkozuba KR, žutenica KR, žutenjak PS, žutinica KO, zlatenca LO
*Tragopogon porrifolius* L.	WA0000066426	L	c	kozja brada (throughout), červej BR, kužjo brada VI
*Urospermum picroide*s (L.) Scop. ex F.W.Schmidt	WA0000066304	L	c	cistacil VI, cistocel VI, lipavica PS, loćika KO, plještika ML, pješti guzica, tustocel VI, tustočen BR, tustočina BR, tutošć BR, tustočel HV, kostočel KO
Boraginaceae				
*Borago officinalis* L.	WA0000066357	L	c	borač LO, boražina LO, buražina SO, buražija KO, krastavac ML
*Echium italicum* L.	WA0000066340	L	c	–
Brassicaceae				
*Bunias erucago* L.	WA0000066909	L	c	pakoleć ML, šurlin KO
*Calepina irregularis* (Asso) Thell.	WA0000066416	L	c	šurlin KO
*Capsella bursa-pastoris* (L.) Medik.	WA0000066371	L	c	prskavica LA, rosomač KO, ščupic SO, šurlin KO
*Diplotaxis* spp.				divlja riga (throughout)
*Diplotaxis muralis* (L.) DC.	WA0000066313	L	r/c	
*Diplotaxis tenuifolia* (L.) DC.	WA0000066337	L	r/c	
*Eruca vesicaria* (L.) Cav.	WA0000066491	L	r	divlja riga PS
*Nasturtium officinale* L.	WA0000066343	L	r/c	kreš PG, kriš KR
*Raphanus raphanistrum* L. s.l.	WA0000071107	L	c	divlja rodakva SO, divlja repa SO, divlja rokva PS
*Sisymbrium officinale* (L.) Scop.	WA0000066418	L	c	drozguja KO
Capparaceae				
*Capparis orientalis* Veill.	WA0000066334	Buds	m	kapar (throughout)
Caryophyllaceae				
*Stellaria media* L.	WA0000066359	L	c	miš(j) akinja KO
*Silene latifolia* Poir.	WA0000066393	L	c	škripac SO, škripavica PS
*Silene vulgaris* (Moench) Garcke	WA0000071139	L	c	učjak PG, uš(l) jak PG, uvce CR, pušina LO
Convolvulaceae				
*Convolvulus arvensis* L. and possibly other species from the genus	ZAGR40001	L	c	slak ML, zlak KO, KR, slačica KO
Cytinaceae				
*Cytinus hypocistis* (L.) L.	Protected species	FL + L	r	prasica PS, kokošica DO
Dioscoreaceae				
*Dioscorea communis* (L.) Caddick & Wilkin	ZAGR39307	SH	c	bljušć, bljušt, blušć, blušt; also: kuke ML, kukolj/kukelj PS, UG
Dipsacaceae				
*Knautia integrifolia* (Honck. ex L.) Bertol.	ZAGR39815	L	c	rešetnica KR
Euphorbiaceae				
*Mercurialis annua* L.	WA0000066409	L	c	prajc VI, prajca VI
Fabaceae				
*Lotus edulis* L.	WA0000066450	IF	r	golubinjica VI, gominjac VI
*Pisum sativum* subsp. *elatius* (M.Bieb.) Asch. & Graebn.	WA0000071112	SH, IF	r	divlji biž VI
*Robinia pseudoacacia* L.	WA0000066466	FL	r/c	akacija, akacia, drača PG, RA, ščavljak ŠO
*Vicia narbonensis* L.	WA0000071113	SH, IF	r	divlji bob VI
Geraniaceae				
*Erodium cicutarium* L.	WA0000071137	L	c	iglica KO, PS
Malvaceae				
*Malva sylvestris* L.	WA0000066400	L	c	sljez VI, sirćić PG
Papaveraceae				
*Papaver rhoeas* L.	WA0000066381	L	r/c	mak, also: papaver VI, ugor glova VI
Plantaginaceae				
*Plantago* spp.				trputac DO, KR, PS, VI, lokvar KR, trbušac KR
*Plantago lanceolata* L.	ZAGR39306	L	c	
*Plantago major* L.	ZAGR39699	L	c	
*Plantago media* L.	ZAGR39712	L	c	
Poaceae				
*Avena sterilis* L.	WA0000066925	L	r	sviralica DO
Polygonaceae				
*Rumex pulcher* L.	ZAGR39692	L	c	kiselica KR, PG, šćav, divlja blitva KR
*Rumex* sp.		L	c	kravlja riljica PG
Portulacaceae				
*Portulaca oleracea* L.	WA0000066314	L	r/c	tucanj DO, PS, RA, UG, SO, tušć BR, HV, UG VI, tušt BR, CR, HV, KO, LO, PG, SO, VI; also: roškan BR, tušanj SO, tustoč BR, HV
Posidoniaceae				
*Posidonia oceanica* (L.) Delile	WA0000066903	Basal part	r	valiga KO, vlasnica VI, vlasinica VI
Ranunculaceae				
*Clematis vitalba* L.	WA0000066476	SH	c	pavitina LO, škrabutina ML, škrebut CR, tertina/trtina/trta KR
Rosaceae				
*Rubus ulmifolius* Scott.	ZAGR39711	SH		drača ML, kupina VI
Rubiaceae				
*Theligonum cynocrambe* L.	WA0000066437	L	c	kokošja jetrica BR
Smilacaceae				
*Smilax aspera* L.	WA0000066325	SH	c	tetevika BR, KO, ML, tetivika LO, tetovica HV, totovika/tutuvika SO, tutuvica VI, jarika PG
Urticaceae				
*Parietaria judaica* L.	WA0000066338	L	c	šćurenica KR, crkvina KO, SO, šćirenica LO, RA
*Urtica* spp.				kopriva ML, RA, SO, UG, LO, VI, SO, KR, LA; also: ožeguja CR, ožigulja CR, už(e) gavica KR, žegavica PS, žiguja, ortika CR, žigavica CR, KR, pokriva KR, žgavica, KR
*Urtica dioica* L.	WA0000066481	L	c	
*Urtica pilulifera* L.	WA0000066441	L	c	
*Urtica urens* L.	WA0000066423	L	c	
Violaceae				
*Viola odorata* L.	WA0000066363	FL	r	ljubičica LO, ljubica KO

Part used—*L* leaf, *WH* whole, *IF* immature fruits, *FL* flowers, *SH* asparagus-like shoots (young vegetative shoots, especially their top part)

Preparation—*r* raw, *c* cooked, *lb*-long baking or boiling, *m* marinated in vinegar

The codes consisting of two letters (in the last column) indicate the first two letters of the name of the studied island, except *DO* for Dugi Otok, *PG* for Pag, *PS* for Pašman and *SO* for Šolta

^a^Used only until the mid-twentieth century

**Table 3:** The diversity of wild vegetables on different islands with the number of interviews in which they were listed (15 interviews were performed in each island)

	ALL	Brač	Cres	Dugi Otok	Hvar	Korčula	Krk	Lastovo	Lošinj	Mljet	Pag	Pašman	Rab	Šolta	Ugljan	Vis
The total number of folk species in 15 interviews		30	18	19	22	46	29	21	26	30	31	26	24	33	18	39
*Allium ampeloprasum* L.	142	14		5	15	13	2	11	9	9	12	13	3	14	7	15
other *Allium* spp. (mainly *Allium roseum* L.)	6	1					2		1			1		1		
*Amaranthus* cf. *retroflexus* L.	6					2	y				2		1	1		
*Anethum graveolens* L.	11	1						3			2			5		
*Apium graveolens* L.	1										1					
*Arum italicum* Mill.	7		2	1			1		1					1		1
*Asparagus acutifolius* L.	173	11	13	6	7	9	14	15	14	11	13	10	12	13	13	12
*Asparagus officinalis* L.	1		1													
*Asphodelus aestivus* Brot.	2								1							1
*Avena sterilis* L.	1			1												
*Bellis* sp.	2					1										2
*Beta vulgaris* L.	28	1	2	1		1	y	4	12	2		1			3	1
*Borago officinalis* L.	13					1			10	1				1		
*Bunias erucago* L.	2					1				1						
*Bunium alpinum* Waldst. & Kit. s.l.	10	1										8			1	
*Calepina irregularis* (Asso) Thell.	2					2										
*Capparis orientalis* Veill.	43	5			6	4		6	2	1	1		1	8	1	8
*Capsella bursa-pastoris* (L.) Medik.	4					2		1						1		
*Carduus pycnocephalus* L.	10			1		4					1		1	2		1
*Chenopodium album* L.	13					1	1	1			5		1		1	3
*Chondrilla juncea* L.	1										1					
*Cichorium intybus* L.	138	3	2	7	11	6	5	13	6	14	14	14	10	9	14	10
*Clematis vitalba* L.	10		1				7		1	1						
*Convolvulus arvensis* L. and possibly other species from the genus	3					1	1			1						
*Crepis dioscoridis* L.	22	3			8	3										8
*Crepis rubra* L.	5	2				3										
*Crepis sancta* (L.) Babc.	4		2			1	y						1			
*Crepis* sp. - other species	8		3		1				1	1	1		1			
*Crithmum maritimum* L.	60	3	1	3	2	5	1	7	10	7	5		1	8	1	6
*Cytinus hypocistis* (L.) L.	6			1								5				
*Daucus carota* L.	13	1				8		1			1	1		1		
*Dioscorea communis* (L.) Caddick & Wilkin	88	8	4	2	1	7	9	7	6	14	10	2	2	8	6	2
*Diplotaxis* spp.	90	7	10	5	2	5	11		9	2	5	4	7	7	6	10
*Diplotaxis muralis* (L.) DC.																
*Diplotaxis tenuifolia* (L.) DC.																
*Echium italicum* L.	1										1					
*Erodium cicutarium* L.	3					1						2				
*Eruca vesicaria* (L.) Cav.	1											1				
unidentified Fabaceae	2						1				1					
*Foeniculum vulgare* Mill.	153	13	4	13	11	14	8	3	10	5	13	12	12	11	11	13
*Helminthotheca echioides* (L.) Holub.	18	1			3	2				1		6	4			1
*Knautia integrifolia* (Honck. ex L.) Bertol.	2						2									
*Lactuca perennis* L.	3	1												1		1
*Lactuca sativa* L. [feral]	3				1	1								1		
*Lactuca serriola* L.	18	2		1	2	4		1		3		2		2		1
*Lactuca viminea* (L.) J. Presl & C.Presl	6					5										1
*Leontodon tuberosus* L.	11	1				7				3						
*Lotus edulis* L.	4															4
*Malva sylvestris* L.	2										1					1
*Mercurialis annua* L.	3					z										3
*Leopoldia comosa* (L.) Parl.	1						1									
*Nasturtium officinale* L.	4						1				3					
*Ornithogalum* sp.	1													1		
*Papaver rhoeas* L.	85	10		5	10	6	4	3		9	2	8	5	11	5	7
*Parietaria judaica* L.	6					1	1		1				1	1		1
*Pisum sativum* subsp. *elatius* (M.Bieb.) Asch. & Graebn.																1
*Plantago* spp.	6			1			3					1				1
*Plantago lanceolata* L.																
*Plantago major* L.																
*Plantago media* L.																
*Portulaca oleracea* L.	55	5	1	8	3	2	4	1	1		2	3	4	7	9	5
*Posidonia oceanica* (L.) Delile	2					y										2
*Raphanus raphanistrum* L. s.l.	5											1		4		
*Reichardia picroides* (L.) Roth	94	12		2	10	13	2	1	9	3	10		7	11	4	10
*Rhagadiolus stellatus* (L.) Gaertn.	1					1										
*Robinia pseudoacacia* L.	2										1		1			
*Rubus ulmifolius* L.	2									1						1
*Rumex pulcher* L.	9					4	1			1	2				1	
*Ruscus aculeatus* L.	18		1				8	3	2	2			2			
*Salsola soda* L.	2										2					
*Sarcocornia fruticosa* (L.) A.J.Scott	8								2				6			
*Scolymus hispanicus* L.	11					2	2				5	1				1
*Scorzonera laciniata* Jacq.	12	4			1	4						1		1		1
*Silene latifolia* Poir.	9		1							1	7					
*Silene vulgaris* (Moench) Garcke	3								1			1		1		
*Silybum marianum* (L.) Gaertn.	1						1									
*Sisymbrium officinale* (L.) Scop.	2					1				1						
*Smilax aspera* L.	10	1			1	1			1	2	1			2		1
*Smyrnium olusatrum* L.	3							3								
*Sonchus* spp.	175	15	9	6	14	14	8	12	10	13	14	14	12	14	6	14
*Sonchus asper* (L.) Hill																
*Sonchus asper* subsp. *glaucescens* (Jord.) Ball ex Ball																
*Sonchus oleraceus* L.																
*Stellaria media* L.	2					2										
*Taraxacum* sp.	43	2	3	2	2	4	12	5	1	5	2	1	1	1	1	1
*Theligonum cynocrambe* L.	1	1				z										
*Tordylium apulum* L.	8					8										
*Tragopogon porrifolius* L.	12	2			2	2				1				4		1
*Urospermum picroide*s (L.) Scop. ex F.W.Schmidt	25	3			5	5				4		2		1		5
*Urtica* spp.	38	8	5				10	4	2	1		1	2	6	5	4
*Urtica dioica* L.																
*Urtica pilulifera* L.																
*Urtica urens* L.																
*Vicia narbonensis* L.	1															1
*Viola odorata* L.	2					1	y		1							

*y* use not recorded in the first interviews (15 per island) but recorded in further field studies, *z* recorded in archival sources [46]

Five taxa, i.e. *Asparagus acutifolius* L., *Cichorium intybus* L., *Dioscorea communis* (L.) Caddick & Wilkin, *Foeniculum vulgare* Mill. and *Sonchus* spp., are gathered to some extent on all the islands, and the collection of *Allium ampeloprasum* L., *Crithmum maritimum* L., *Diplotaxis* spp., *Papaver rhoeas* L., *Portulaca oleracea* L., and *Taraxacum* spp. is or was practiced on all but one or two islands.

**Fig. 3:**
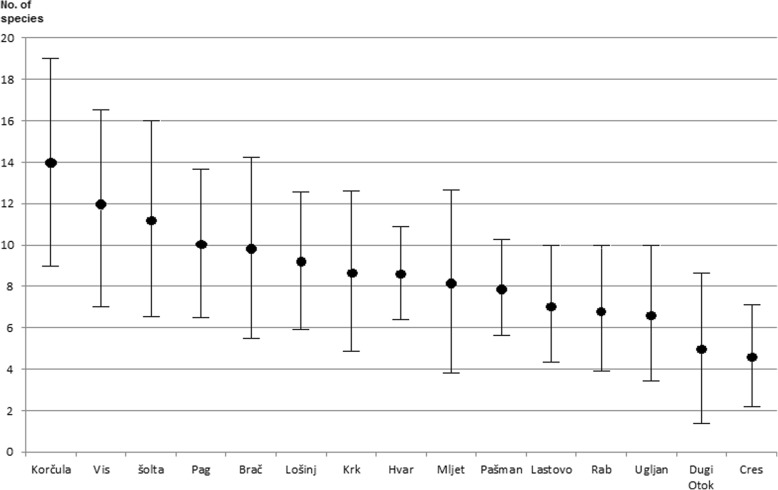
Mean number (and standard deviation) of wild vegetable species mentioned per interview

The studied patterns of wild vegetable use were relatively weak (Table 4, Fig. 3), and no significant correlations (*p* < 0.05) between independent and dependent variables were found (although a few approached the significance level). Thus, all the hypotheses can be rejected from the statistical point of view. The highest correlations were found between geographical longitude and the number of wild vegetable species, and between population size and the number of wild vegetable species. Surprisingly, the total number of species in the flora showed a negative correlation with the median number of vegetable species used. Correlations between the total number of wild vegetable species and independent variables were nearly identical, as the total number of vegetable species per island and the median number of vegetables listed were highly correlated. The island’s degree of isolation from the mainland and its area seemed to have negligible effects on the median wild vegetable number listed. We must bear in mind that the above-discussed correlations are statistically not significant. The question rises whether the results would be significant or different if a larger number of respondents were studied. The answer is probably ‘not’, as we think that the 15 interviews we did for each island were very representative. This is supported by data from two islands from which we have more interviews. In the largest and most populated island, Krk, 55 interviews were conducted altogether [21] and 33 species of wild vegetables were recorded, whereas in the first 15 interviews selected for this study, 29 were found. However, the effect of some of the independent variables (area, population, flora) might have been stronger if islands smaller than 40 km^2^ had been included.

**Table 4:** The correlation matrix of all the variables in the study (correlation coefficients in the lower left half, *P* values in the upper right half). Most correlations are expressed as Pearson *r* coefficient. Only correlations for area (printed in italics) were calculated using Spearman *r*s rank correlation coefficient (see explanation in the ‘Methods’ section)

	No. of veg	Median no. of veg	Area^a^	Population	Flora	Longitude	Isolation^b^
No. of veg		2.6E−05	0.75	0.21	0.37	0.11	0.57
Median no. of veg	0.87		0.96	0.15	0.48	0.06	0.67
Area^a^	*0.09*	*− 0.01*		− 0.03	0.07	0.45	0.15
Population	0.34	0.39	*0.55*		0.09	0.73	0.01
Flora	− 0.26	− 0.21	*0.49*	0.47		0.14	0.55
Longitude	0.42	0.50	*− 0.17*	− 0.10	− 0.42		0.47
Isolation^b^	− 0.15	− 0.12	*− 0.39*	− 0.63	− 0.18	0.20	

^a^For this variable, Spearman *r*s (rank correlation) coefficient was calculated

^b^This variable was log-transformed in order to achieve normal distribution

**Fig. 4:**
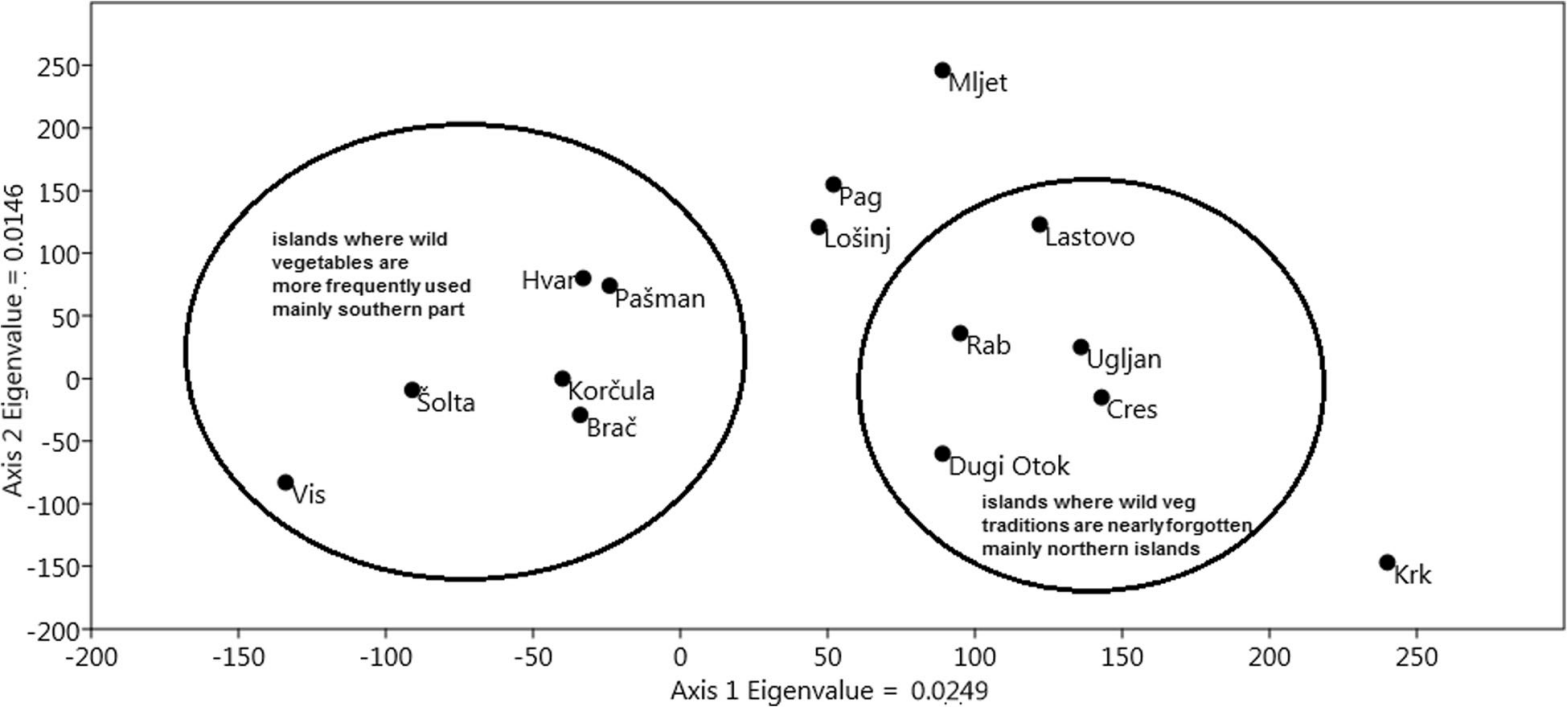
Results of DCA analysis

Surprisingly, geographical location expressed by longitude was most strongly correlated with wild vegetable species richness. This indicates that a larger scale pattern of increasing wild vegetable ‘popularity’ going from the northeastern Adriatic southeastwards towards Dubrovnik is stronger than island biogeography effects. A similar southeastward pattern was earlier detected for the richness of wild vegetables sold in the markets along the main coast of Croatia [19]. The spatial distribution of islands on the two main axes of DCA analysis corresponded to some extent to their geographical position. All the central Dalmatian islands (i.e. Šolta, Brač, Hvar and Vis) created one cluster together with Korčula and Pašman (which is the closest to them from all the Zadar Archipelago islands). Most islands of the Zadar Archipelago (i.e. north Dalmatian islands—Pag, Dugi Otok and Ugljan) were clustered together with the islands of the Kvarner Archipelago (Cres, Krk and Rab). Krk and the central Dalmatian Vis were most distant from other islands and formed two opposite sides of the diagram (Fig. 4).

**Fig. 5:**
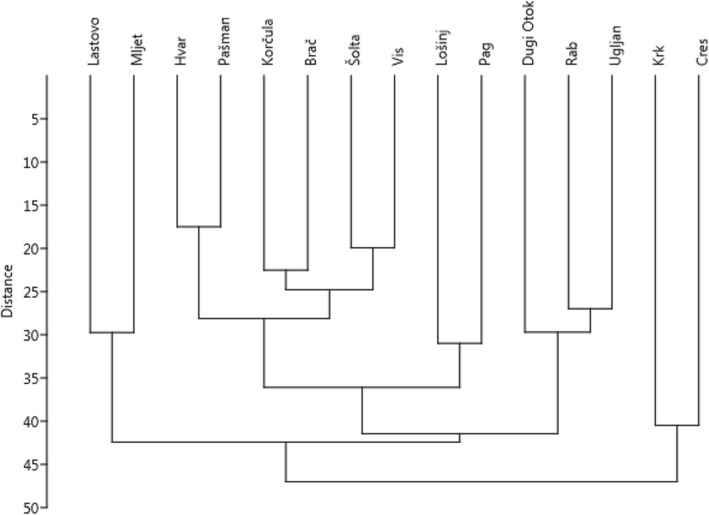
The dendrogram of UPGMA clustering of islands based on the matrix of wild vegetables used in them

The dendrogram from UPGMA clustering (Fig. 5) shows similar results to the DCA analysis. Here, all the central Dalmatian islands (i.e. Šolta, Brač, Hvar and Vis) created one cluster together with Korčula and Pašman. As this cluster also contains the islands with the strongest use of wild vegetables, we could say that this area now constitutes the core region in which knowledge is preserved in the Adriatic, whereas the ‘peripheral’ islands north and south of it are those where wild vegetables have been forgotten to a greater extent.

The island biogeography theory [2] states that the species diversity of islands is positively correlated with the island size and negatively correlated with its distance from the mainland. It is striking that vascular floras are negatively correlated (though again not significantly) with wild vegetable diversity. It is probably caused by the fact that most wild vegetables are ruderal weeds, which may thrive better in anthropogenic degraded habitats rather than on natural islands with better preserved (semi-) natural vegetation.

**Fig. 6:**
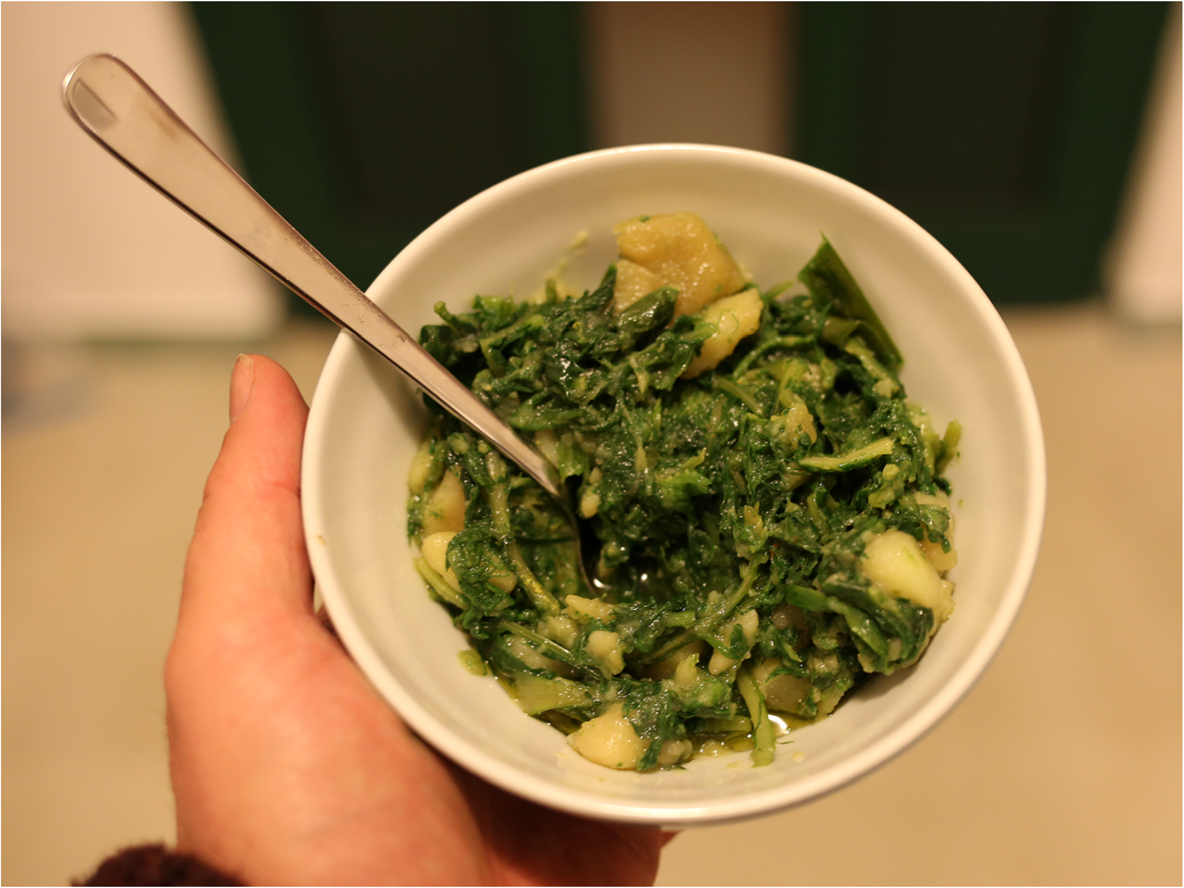
A bowl of *gruda*, i.e. wild vegetable mix from Vela Luka, Korčula, cooked with potatoes and spiced with olive oil and salt

Although the wild vegetables used on the islands are very similar to those on the main coast of Croatia, their preparation differs slightly (Fig. 5). On the islands, people tend to cook the wild vegetables only for a short time or eat them raw, whereas on the mainland, the vegetable mix is often cooked for 20–30 min [19–22]. *Asparagus* spp. and asparagus-like plants are usually prepared separately, boiled or fried and eaten with eggs. Tender, bitter *Asteraceae*, such as *Cichorium*, *Crepis* and *Taraxacum*, as well as *Diplotaxis*, *Portulaca* and *Papaver*, are eaten raw or only briefly boiled. *C. maritimum* shoots and *Capparis orientalis* Veill. flower buds are marinated in home-made wine vinegar. Other species are usually mixed and boiled. Wild vegetables are often cooked with one or two potatoes and served with plenty of olive oil (Fig. 6). The mixed wild vegetables are usually called interchangeably *divlje zelje* (literally ‘wild herbs’) or *mišanca* (literally ‘mixture’), with small phonetic variants of these names depending on the dialect of the particular settlement. For example, on the island of Rab in Palit, we recorded the name *mišancija*, in Banjol *mišjanca*, and *mješanca* in the town of Rab. The largest variety of names for the mixture occurs on Brač with *parić* in Sumartin, *parež* in Gornji Humac, *porež* in Pražnice, Pučišća and Škrip, *divljač* in Pražnice, *poreč* in Nerežišče, *pareš* in Bol, *divjo zelje* in Dračevica, Mirca and Milna, and *mišancja* in Gornji Humac. Around the town of Cres (island Cres), the names are *divljina* or the Italian word *erbate.* On the island of Korčula, the western part (Vela Luka and Blato) uses the name *gruda* and the eastern part (e.g. Čara and Žrnovo) uses the name *parapač. Pakojeć* is the name used on Lastovo, whereas on Mljet it is called *pakoleć* and *podparuša*. On Vis, it is called *gorko*/*gorku zelje* (literally ‘bitter herb’) or *divjo*/*divlju zelje.*

Although we recorded a long list of wild vegetables used in the archipelago as a whole, the use of this category of food has dramatically declined. On some islands, such as Cres or Lastovo, the list of plants used must already have been quite short a few decades ago, but on some islands such as Brač, Šolta, Vis or Pašman, the collecting of wild vegetables was widespread even at the end of the twentieth century and collapsed quite recently, with several older people still practicing it now. It is only Korčula where the custom is important even nowadays, although signs of the deterioration of knowledge and a reduction in the number of collected species are visible even there. The differences between islands cannot be explained based on apparency [40] or resource availability theory [41] of the main species of wild vegetables as they are common and easily found on each island. They are probably caused mainly by the differences in the rate of abandonment of the old tradition of eating wild vegetables. The gradual abandonment of using wild vegetables has been observed in other Mediterranean regions of Europe (see e.g. [26, 42]), and it is only recently that some health-conscious people and those interested in cooking have gone back to it [17, 42]. We recorded only two species for which some of our respondents observed a positive trend. One of them is *Asparagus acutifolius.* Around 30–50 years ago, in some villages, it was not collected, but the use of this species spread to most families. Many informants attribute this increase in consumption to the cessation of grazing by livestock and a consequent increase in the populations of *A. acutifolius* in the wild. The other is *C. maritimum* which was collected in the past but to a much lesser extent. Its current widespread use has been popularised both by TV cooking programmes and by people from outside the community being seen to collect it. *C. maritimum* is now commonly sold preserved in vinegar as a souvenir for tourists.

Why has the population of Korčula preserved the largest number of wild vegetables used? Korčula was spotted as a place with a rich tradition of using wild vegetables as far back as 1981, when a TV programme broadcast by ‘Radiotelevision Zagreb’ was made (after [25]). An article about the tradition was also written by a local museum worker [43], and a book about plant uses was compiled by a local school teacher together with her primary school students [44]. Long lists of wild vegetables used also occur in the local dialect dictionary [45] and in a monograph of the island [46]. No other studied island has produced such publications or such a strong local identification with using wild vegetables. There may be another reason for the very robust knowledge of wild vegetables in Korčula. The island has always had a large population which maintains its subsistence on cultivated crops. In the early twentieth century, Korčula experienced a very severe famine, as a consequence of the mass destruction of vineyards caused by a phylloxera epidemic (information from older informants). In contrast to this, less populated and more isolated islands such as Dugi Otok and Lastovo could base their nutritional economy on marine resources and were not affected by malnutrition—the latter also served as a smuggling base, which brought high cash profits.

The list of wild vegetables used is very typical for the Mediterranean areas of Europe [15, 16, 26, 42, 47–56]. It is also similar to those recorded in other parts of Croatia [19–24].

Some of the islands, especially in the north-western half of the study area (Kvarner and Zadar archipelago), contain large salt-marshes. Surprisingly, the only typically coastal halophilous plant widely utilised in the Adriatic Islands is *C. maritimum.* We only found a few respondents using wild *Beta vulgaris* L. (on various islands throughout), *Sarcocornia fruticosa* (L.) A.J.Scott. (only on Rab and Lošinj) and *Salsola soda* L. (on Pag and some smaller islands between Zadar and Split not included in this study). Our data show that coastal areas were treated as sources of food for animal stock, rather than sources of plant food for humans. *C. maritimum* is now widely collected for pickles, but in most cases, this is a new fashion which people took up a few years ago, though a certain proportion of informants remember making such pickles in their childhood as well. The lack of food use of sea marsh plants is particularly striking on Pag, famous for this type of vegetation.

Out of the recorded genera, we have not found any whose use is specific only to the Adriatic Islands apart from seagrass (*Posidonia oceanica* (L.) Delile). The basal parts of the shoots of this monocot species were used to be eaten as a snack on Korčula and Vis. Unfortunately, no traces of the traditional use of seaweeds have been recorded. Another interesting find was the custom of eating raw tubers of *Bunium alpinum* Waldst. & Kit. s.l. on Pašman and Brač. We have not found any other food uses of these two species in world literature, though other *Bunium* species are widely known to be used as food. For example, in Spain, three species are used, *Bunium balearicum* (Sennen) Mateo & López Udías, *Bunium macuca* Boiss and *Bunium pachypodum* P.W. Ball [40]. Yet, another interesting tradition is eating the flowering shoots of the parasitic *Cytinus hypocistis* (L.) L., which is still widely known (though its practice ceased a few decades ago) on the island of Pašman. The consumption of *C. hypocystis* was reported before only from small localities in Spain, Portugal, Turkey and Greece [46–50].

Vis is the island with the second longest list of wild vegetables used. What differentiates it from other islands is the custom of eating young shoots and green pods of a few Fabaceae plants, with the immature fruits of *Lotus edulis* L. particularly prized as a raw snack or for pickling (like capers).

## Conclusions

The recorded relationships between the demographic and geographical features of islands were weak and statistically not significant. It is most likely that cultural and historical factors diversifying the use of plants in particular islands are stronger than the above-mentioned quantitatively measurable variables. A general trend of increasing richness in wild vegetables from north-west to south-east (which can have cultural or historical reasons) can be observed. More ethnobotanical quantitative studies on islands are needed to form an ‘island ethnobotany theory’.
